# *Smilax glabra* Flavonoids Inhibit AMPK Activation and Induce Ferroptosis in Obesity-Associated Colorectal Cancer

**DOI:** 10.3390/ijms26062476

**Published:** 2025-03-10

**Authors:** Jianqin Xu, Zhaowei Cai, Ziyao Pang, Jiayan Chen, Keyan Zhu, Dejun Wang, Jue Tu

**Affiliations:** 1 Laboratory Animal Research Center, Academy of Chinese Medical Sciences, Zhejiang Chinese Medical University, Hangzhou 310053, China; 13958083242@163.com (J.X.); zwcai@zcmu.edu.cn (Z.C.); pang3056527363@163.com (Z.P.); 18407878185@163.com (J.C.); zjzygyz@163.com (K.Z.); 2 School of Pharmacy, Zhejiang Chinese Medical University, Hangzhou 310053, China

**Keywords:** *Smilax glabra* flavonoids, AMPK activation, ferroptosis, obesity-associated CRC

## Abstract

*Smilax glabra* flavonoids (SGF), the active components of *Smilax glabra* Roxb., have been demonstrated to exhibit antioxidant activity and metabolic benefits in obesity, leading us to further explore their antitumor effects in obesity-related colorectal cancer (CRC). This study investigated the antiproliferative effects of SGF on obesity-related CRC by using a murine colon adenocarcinoma MC38 cell line. The underlying mechanisms were further explored via RNA-Seq and bioinformatics analysis in combination with experimental validation. SGF was proven to possess cytotoxic effects against MC38 cells, indicated by the inhibition of proliferation and migration, especially in an adipocyte-rich environment. In line with this, SGF exhibited much stronger antiproliferative effects on MC38-transplanted tumors in obese mice. Transcriptomics analysis showed that the cytotoxic effects of SGF might be related to the AMPK pathway and ferroptosis. On this basis, SGF was confirmed to induce ferroptosis and dictate ferroptosis sensitivity in a high-fat context mimicked by a two-step conditioned medium (CM) transfer experiment or a Transwell coculture system. The results of Western blotting validated that SGF suppressed the phosphorylation of AMPK, accompanied by alterations in the biomarkers of ferroptosis. These results demonstrate that SGF exerts in vitro and in vivo antiproliferative effects in obesity-associated CRC through inhibiting AMPK activation, thereby driving ferroptosis.

## 1. Introduction

Colorectal cancer (CRC) is the third most common cancer and ranks second in cancer-related deaths worldwide [1]. The number of new cases and deaths from CRC is increasing rapidly, such that it is predicted to exceed 2.2 million new cases and 1.1 million deaths by 2030 [2]. Despite the benefits of early screening, surgery, and other therapeutic interventions, the five-year relative survival rate of CRC patients has not changed significantly in the past few decades [3]. Obesity is identified as a leading risk factor for more than 13 types of cancer, including CRC, according to emerging epidemiological studies [4,5]. It has been revealed that the CRC incidence rises by 7% for every 2.4 unit increase in body mass index (BMI) [6,7]. Moreover, obesity is associated with a poor prognosis and advanced clinicopathological status in CRC patients [8]. In addition, obese CRC patients are prone to develop resistance to target reagents and chemotherapies widely used in the clinic [9]. Therefore, it is crucial to explore effective prevention and treatment approaches that are specific to obesity-associated CRC.

Previous studies indicate that obesity promotes cancer by accumulating adipose tissue, thus leading to systemic changes in steroid hormones and adipokine production, metabolic disorders, chronic subclinical inflammation, and alterations of the gut microbiome [10,11,12]. The latest studies propose that hypertrophic adipocytes constitute a major cell type that is abundantly associated with tumor cells in the tumor microenvironment (TME), playing an important role in cancer progression [13]. Consistently, obesity-related cancers including CRC are always located adjacent to the adipose tissue, and metastatic colon cancer cells often encounter adipocytes as they first disseminate from their primary tumor site [14]. Mechanistically, the direct interaction of tumor cells with surrounding adipocytes plays a positive role in the tumorigenic progress. Emerging data show that lipids produced in adipocytes could be transferred to cancer cells to promote tumor growth, especially in nutrient deprivation conditions [15,16,17,18]. Meanwhile, tumor cells stimulate the release of fatty acids by promoting lipolysis in adipocytes, indicating two-way communication between cancer cells and adipocytes in the TME [14]. Overall, the tumor–adipocyte microenvironment might provide new therapeutic targets and pathways for obesity-related CRC.

*Smilax glabra* Roxb. (SG), the dry rhizome of Smilacaceae, is referred to as tu fuling in Chinese medicine. It has been widely used as a food and folk medicine in many countries due to its various beneficial properties [19]. Modern pharmacological studies have shown that SG rhizome extracts have a variety of pharmacological properties, including anti-infective [20], immunomodulatory [21], antioxidant [22], and hepatoprotective [23] properties. Moreover, the antitumor activity of SG and its bioactive components has been suggested regarding several types of cancer, such as hepatocarcinoma [24], breast cancer [25], and prostate cancer [26]. Our previous investigations revealed that the flavonoids of *Smilax glabra* Roxb. (SGF) exhibited anti-cardiac hypertrophy effects by regulating calcium-related signaling pathways [27] and inhibiting the Raf/MEK/ERK pathway [28]. Moreover, it was demonstrated that SGF had potent effects against kidney fibrosis [29]. A recent study showed that the flavonoid groups of *Smilax glabra* Roxb. had antitumor immune-regulatory potential in MMTV-PyMT mice [30]. However, the actual anticancer effects and relevant mechanisms of flavonoids from SG in CRC, especially in obesity-related CRC, remain unknown. In our latest study, we proposed the potential metabolic benefits of SGF on human obesity [31], which is in line with the results of previous studies in other metabolic diseases [32], suggesting that SGF might have a unique effect in treating obesity-associated CRC. In the present study, we firstly investigated the antitumor effects of SGF against CRC and obesity-related CRC in vitro and in vivo. SGF was proven to possess much higher cytotoxic efficiency towards obesity-associated CRC. Furthermore, the underlying mechanisms of the cytotoxic action of SGF were explored via RNA-Seq and bioinformatics analysis in combination with experimental validation.

## 2. Results

### 2.1. SGF Exhibited Cytotoxic Effects Towards Obesity-Associated Colorectal Cancer

**Figure 1:**
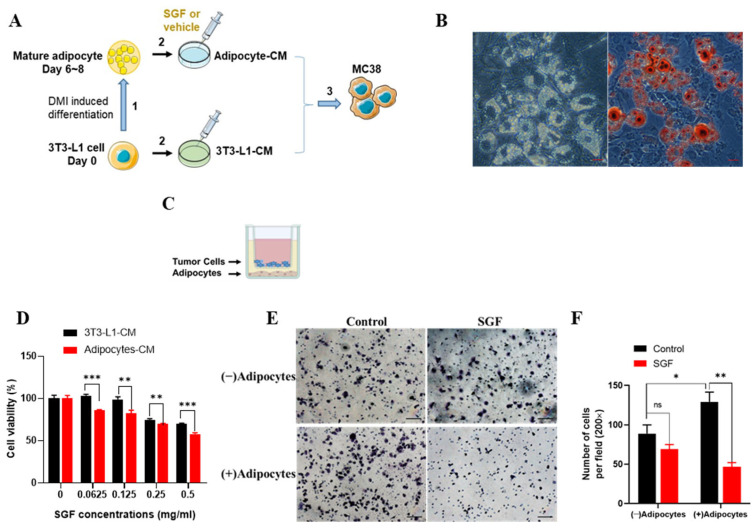
SGF inhibited the proliferation and migration of MC38 cells in the adipose microenvironment. (**A**) Flowchart of conditioned medium (CM) preparation and treatment of MC38 cells. (1) Differentiation of pre-adipocytes to mature adipocytes; (2) preparation of CM from differentiated mature adipocytes and 3T3-L1 fibroblasts; (3) treatment of tumor cells with the corresponding CM in the presence or absence of SGF. (**B**) Morphological observation (left) and ORO staining (right) of 3T3-L1 adipocytes induced with DMI medium until differentiation at day 8. Images were captured at 40× magnification. Scale bars, 10 μm. (**C**) Cartoon of tumor cell and adipocyte Transwell coculture assay. (**D**) The cell viabilities of MC38 cells treated with SGF at the indicated concentrations for 24 h in the corresponding CM via MTT assay. (**E**) Transwell migration assay was performed to detect MC38 cell migration after 0.125 mg/mL SGF treatment in a Transwell coculture system for 72 h and (**F**) the number of migrating cells per field was determined. Scale bars, 100 µm. Data are expressed as mean ± SD (*n* = 5). * *p* < 0.05, ** *p* < 0.01, *** *p* < 0.001 and ns: no significance vs. control.

The conditioned medium (CM) of mature adipocytes or an in vitro Transwell system was utilized to mimic the adipose tissue microenvironment in obesity-associated CRC (Figure 1A,C). Consistent with our previous report [31], mature adipocytes were differentiated from 3T3-L1 cells for 6 to 8 days via the DMI assay. The development of an adipocyte phenotype was identified by Oil Red O (ORO) staining (Figure 1B). Subsequently, the CM from white adipocytes (adipocyte-CM) and 3T3-L1 pre-adipocytes (3T3-L1-CM) were collected to treat three colorectal cancer cell lines, including murine colorectal carcinoma MC38 and CT26, as well as human colon cancer HCT116, with or without SGF treatment (Figure 1A and Figure S1A). The cell viability was determined by MTT assays. As shown in Figure 1D, SGF effectively inhibited the proliferation of MC38 cells in both CM, with adipocyte-CM significantly enhancing its cytotoxic efficacy. This enhanced antiproliferative activity of SGF in the presence of adipocyte-CM was consistently observed in two additional CRC cell lines (Figure S1B,C). Similarly, the Transwell coculture with adipocytes (Figure 1C) promoted the migration of MC38 cells, and 0.125 mg/mL SGF could obviously inhibit their migratory abilities. Meanwhile, SGF at the same concentration had no effect on the migration of MC38 cells in the coculture system without adipocytes in the bottom chamber (Figure 1E,F). These results suggest that SGF possesses much higher cytotoxic efficiency towards obesity-associated CRC.

### 2.2. SGF Inhibited the Growth of MC38 Allografts in Obese Mice

**Figure 2:**
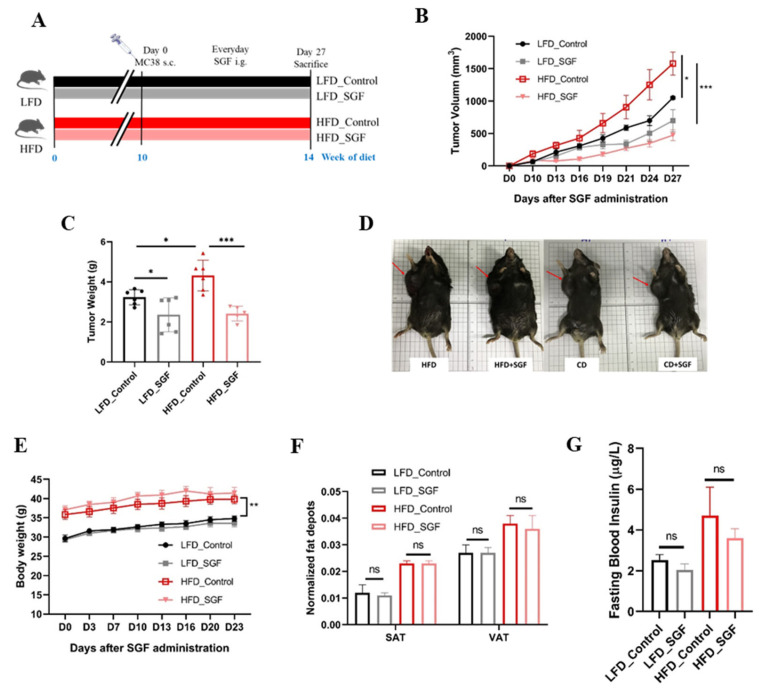
SGF inhibited the tumor growth of MC38 allografts in HFD-fed mice. (**A**) Schematic illustrating animal experimental approach. s.c., subcutaneous; i.g., intragastric. (**B**) Growth curve of MC38-transplanted tumors in mice over time when receiving vehicle or SGF (62.5 mg/kg/day) following 10 weeks of dietary treatment. (**C**) Average final tumor weights of each group. (**D**) Representative photos of tumor-bearing mice after SGF treatment. (**E**) Body weights of tumor-bearing mice. (**F**) Subcutaneous adipose tissue (SAT) and visceral adipose tissue (VAT) were weighed and normalized to body weight. (**G**) Blood insulin levels were measured following a 10 h fast after 14 weeks of dietary treatment. The data are expressed as means ± SEM (*n* = 6). * *p* < 0.05, ** *p* < 0.01, and *** *p* < 0.001 and ns: no significance vs. control.

A syngeneic subcutaneous tumor model using a colorectal adenocarcinoma cell line, MC38, was initially applied to identify the effects of diet-induced obesity on tumor growth and especially the antitumor efficacy of SGF (Figure 2A). After 10 weeks of diet feeding, HFD-fed mice gained more body weight and exhibited systemic obesity-associated metabolic changes, including hyperglycemia, hyperinsulinemia, and hyperlipidemia, suggesting the achievement of an obese phenotype (Figure S2A–F). Then, the mice were subcutaneously injected with cancer cells, which were allowed to grow for 27 days. As expected, our data showed that tumors grew more quickly in the HFD-induced mice than in the LFD groups. Surprisingly, although SGF treatment seemed to attenuate tumor growth in both diet groups, its antitumor effects were remarkedly enhanced in HFD-fed mice (Figure 2B–D). Meanwhile, treatment with SGF for 4 weeks did not ameliorate the obesity parameters of HFD-fed mice (Figure 2E–G), which suggested that the excellent antitumor effect of SGF in obese mice was not merely due to its beneficial effects on the obesity status. These data support the paradox whereby obesity can enhance both tumor growth and the response to SGF; meanwhile, they emphasize that SGF could exhibit much higher antitumor efficiency in obesity-associated CRC.

### 2.3. Transcripome Analysis Suggested the Involvement of the AMPK and Ferroptosis Pathways in Tumors Treated with SGF

**Figure 3:**
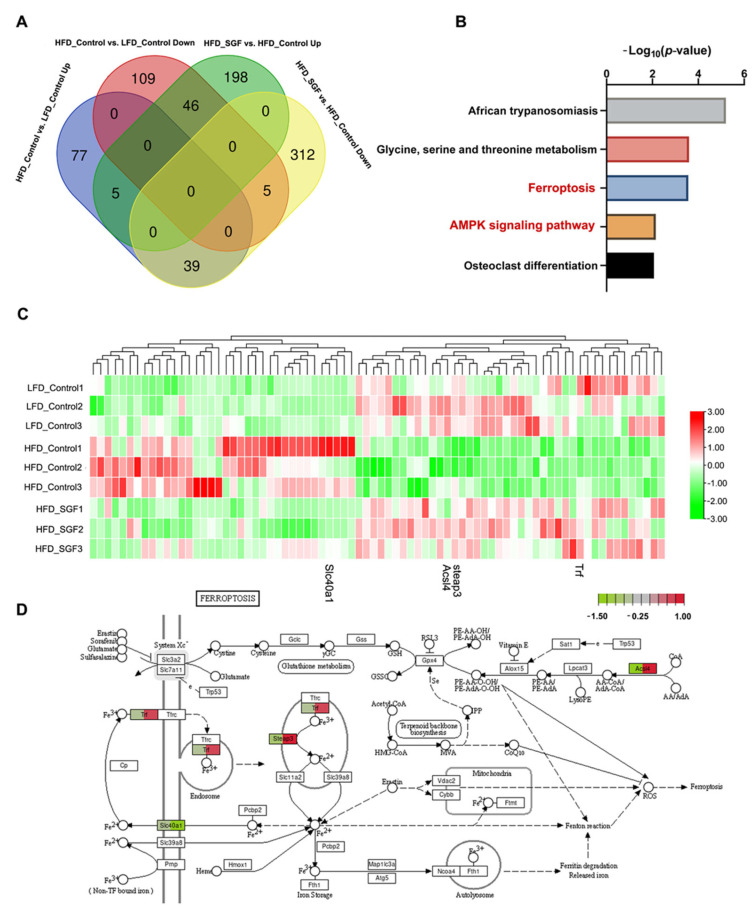
Transcriptome analysis suggests that SGF regulates the expression of genes associated with ferroptosis. (**A**) Venn diagram of DEGs in respective groups. (**B**) KEGG analysis of DEGs altered in HFD group and reversed in HFD_SGF group. (**C**) Heatmap of hierarchical clustering of DEGs between LFD, HFD, and HFD_SGF groups. (**D**) Differences in tumor gene expression in ferroptosis pathway according to KEGG. Red indicates upregulation and green downregulation.

To clarify the molecular mechanisms of the antitumor action of SGF against obesity-associated colorectal cancer, a comprehensive analysis of RNA sequencing data from MC38-transplanted tumors was performed. There were 281 DEGs in the HFD group compared to the LFD group (121 upregulated and 160 downregulated) and 605 DEGs in the HFD_SGF group compared to the HFD group (249 upregulated and 356 downregulated), as shown in Figure 3A. To gain further insights into the functional implications of these DEGs, we selected the genes that were altered in the HFD group while being reversed by SGF in the HFD_SGF group for further analysis, and a cluster analysis of these DEGs was performed using heatmaps (Figure 3C). The results of the KEGG pathway analysis showed the significant enrichment of pathways related to African trypanosomiasis; glycine, serine, and threonine metabolism; ferroptosis; the AMPK signaling pathway; and osteoclast differentiation (Figure 3B). To examine the detailed pathway of ferroptosis and the relative differences in the genes affected by HFD and SGF in CRC, a putative pathway of ferroptosis was constructed based on KEGG mapping (Figure 3D). Genes involved in the regulation of iron uptake and lipid peroxidation (Trf, Steap3, and ACSL4) were decreased in HFD mice but restored in HFD_SGF mice. These data indicated that ferroptosis and the AMPK pathway might be involved in the cytotoxic effects of SGF towards obesity-associated CRC.

### 2.4. SGF Induced Ferroptosis and Dictated Ferroptosis Sensitivity in MC38 Cells in a High-Fat Environment

**Figure 4:**
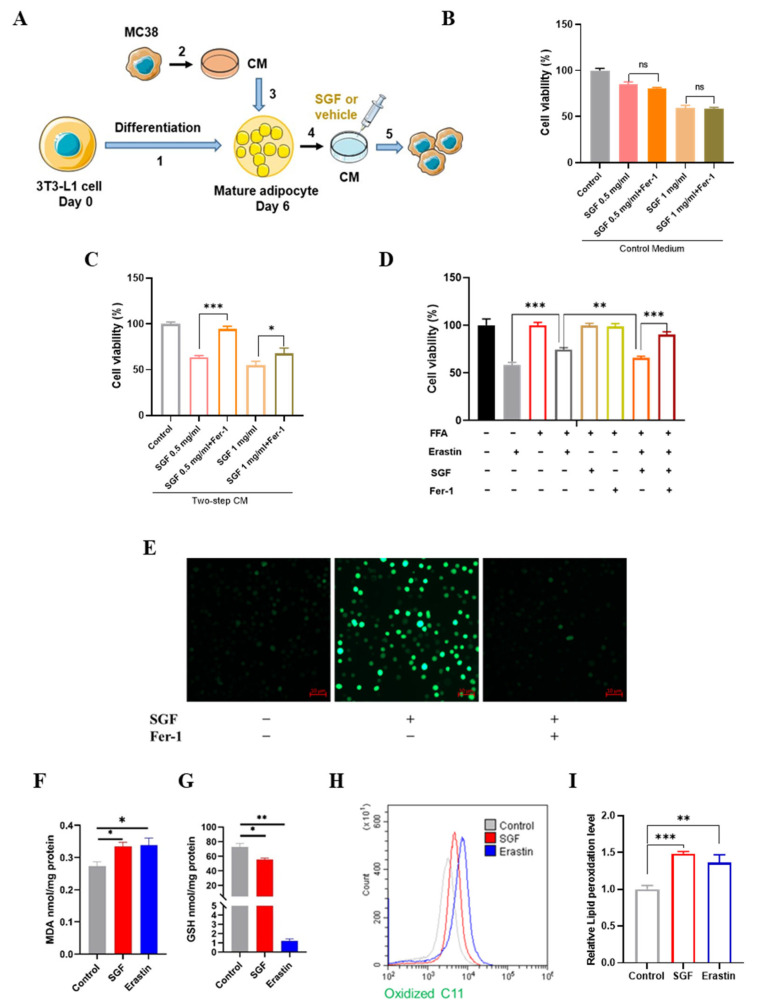
SGF triggered ferroptosis and sensitized MC38 cells to ferroptosis in a high-fat environment. (**A**) Flowchart of two-step CM preparation and treatment of MC38 cells. (1) Differentiation of 3T3-L1 cells to mature adipocytes; (2) preparation of CM from MC38 cells; (3) treatment of differentiated adipocytes with MC38 CM; (4) after exposure to MC38 CM for 24 h, the adipocytes were washed and the medium was changed; (5) after another 24 h, the adipocyte-conditioned media were collected and applied to fresh cultures of MC38 cells in the presence or absence of SGF. (**B**,**C**) After pre-incubation with or without Fer-1 (1 µM) for 60 min, MC38 cells were freshly cultured with control medium (**B**) or two-step CM (**C**) containing SGF (0 mg/mL, 0.5 mg/mL, and 1 mg/mL) for 48 h. The cell viabilities were detected via the MTT assay. The data are expressed as means ± SD (*n* = 3) (* *p* < 0.05, *** *p* < 0.001, and ns: no significance). (**D**) MC38 cells were treated or left untreated with 0.1 mM oleic acid or fatty acid + 2 μM Fer-1 for 60 min. Subsequently, cells were treated simultaneously with fatty acids with 2 μM Erastin in the presence or absence of 0.125 mg/mL SGF for 48 h, and the cell viability was evaluated using the MTT assay. The data are expressed as means ± SD (*n* = 3) (** *p* < 0.01 and *** *p* < 0.001). (**E**) MC38 cells were cocultured with differentiated mature adipocytes in a Transwell system. After being pretreated with or without Fer-1 for 60 min, MC38 cells were incubated with 0.5 mg/mL SGF or a vehicle for 48 h. Following the treatment, MC38 cells were observed for intracellular ROS under a fluorescence microscope by DCFH-DA staining. Scale bars: 10 µm. (**F**,**G**) MC38 cells in a Transwell coculture system were treated with SGF (0.5 mg/mL) and Erastin (10 µM), respectively, for 48 h, and MDA (**F**) and GSH (**G**) production was examined by colorimetry. (**H**,**I**) Lipid peroxidation levels were quantified with BODIPY-C11 using flow cytometry. Red and green fluorescence indicated non-oxidized and oxidized lipids, respectively. Bar graph showing relative levels of lipid ROS in the indicated cells. The data are expressed as means ± SEM (*n* = 3) (* *p* < 0.05, ** *p* < 0.01, and *** *p* < 0.001 vs. control).

According to a study reported previously [33], a two-step CM transfer experiment was designed to mimic the putative bidirectional communication between CRC cells and adipocytes in the tumor–fat microenvironment (Figure 4A). On this basis, Ferrostatin-1 (Fer-1), a ferroptosis-specific inhibitor, was applied to determine whether ferroptosis is responsible for SGF-mediated tumor suppression. As expected, pretreatment with Fer-1 significantly reversed the decrease in the viability of the MC38 cells induced via SGF in the two-step CM (Figure 4C), indicating that ferroptosis contributed to the cytotoxicity of SGF towards obesity-related CRC cells. Interestingly, we noticed that Fer-1 failed to protect against SGF-induced MC38 cell death in the control CM (Figure 4B), which suggests that a high-fat environment might be essential for SGF to exert a pro-ferroptotic effect.

It has been demonstrated that lipids could protect colorectal cancer cell lines against ferroptosis [34]. Given this, the sensitivity to ferroptosis was further observed in MC38 cells. Consistently, 2 µM Erastin, a classic ferroptosis inducer, remarkably reduced the viability of MC38 cells, whereas the suppression was significantly attenuated in the presence of fatty acids. However, combined treatment with Erastin and SGF dramatically recovered the cytotoxic effects, which could be markedly negated by Fer-1 (Figure 4D), suggesting that SGF could sensitize MC38 cells to ferroptosis in a high-fat environment.

To further confirm these notions, a tumor cell–adipocyte Transwell coculture system was applied, and the intracellular ROS level, an indirect hallmark of ferroptosis [35], was examined with DCFH-DA in MC38 cells. SGF notably increased the intracellular ROS levels as represented via the fluorescence intensity of DCF, and pretreatment with Fer-1 almost eliminated these actions (Figure 4E). Then, MDA, which is an aldehyde secondary product of lipid peroxidation, and GSH, which is critical in detoxification after lipid peroxidation, were evaluated using commercial kits. Simultaneously, the accumulation of lipid ROS, a hallmark of ferroptosis, was measured by fluorescent probe C11-BODIPY 581/591 staining followed by flow cytometry. As shown in Figure 4F–I, SGF notably promoted MDA production and depleted GSH in MC38 cells, which was consistent with the action of Erastin as a positive control. In addition, both SGF and Erastin led to the significant accumulation of lipid ROS, as evidenced by the clear shift in the fluorescence signal from red to green. Taken together, ferroptosis is indeed involved in SGF-induced cell death and might be responsible for its distinctive antitumor efficacy in obesity-associated CRC.

### 2.5. SGF Suppressed AMPK Activation, Contributing to Ferroptosis in Obesity-Associated CRC

**Figure 5:**
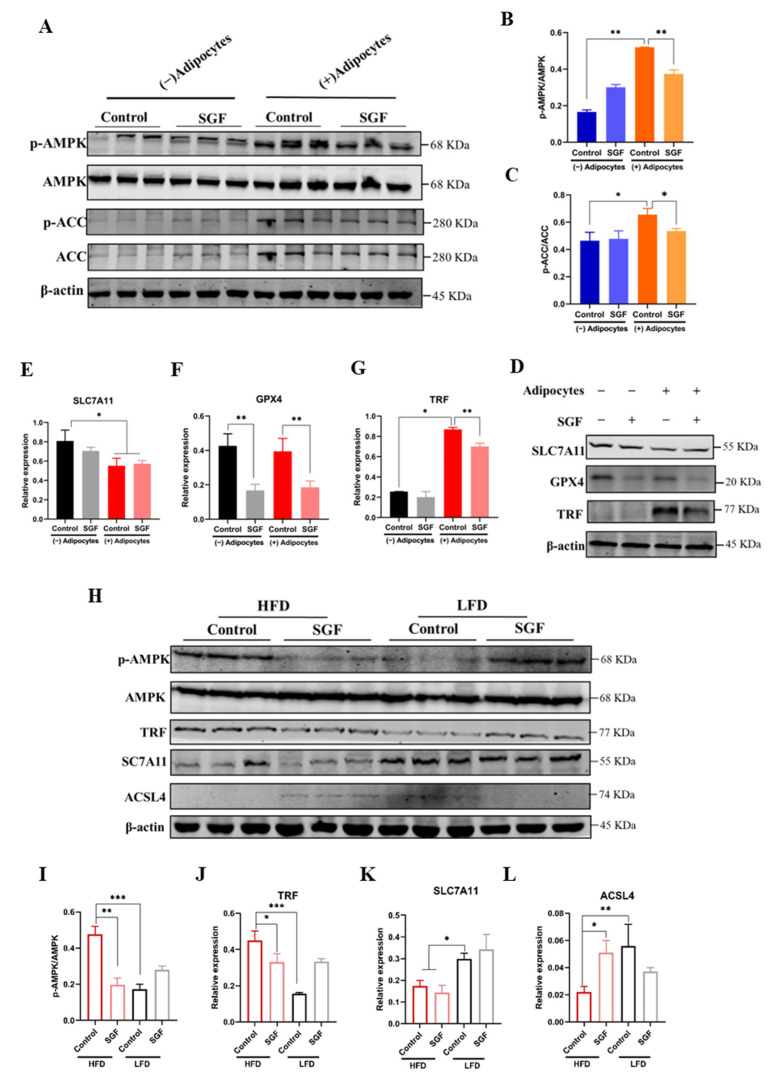
SGF repressed AMPK activation, thus altering cells’ sensitivity to ferroptosis. (**A**–**C**) MC38 cells were cocultured in the presence or absence of adipocytes and treated with SGF (0.5 mg/mL) for 24 h. The phosphorylation of AMPK and ACC was detected via Western blotting. The blots (**A**) are representative of three repeats. The data (**B**,**C**) are expressed as means ± SD (*n* = 3). * *p* < 0.05 and ** *p* < 0.01 vs. control. (**D**–**G**) An immunoblot of ferroptotic protein expression in MC38 cells as indicated. β-actin was used as an equal loading control and blots are representative of three repeats. * *p* < 0.05, ** *p* < 0.01 vs. control. (**H**,**I**) The tumor tissue of the MC38-transplanted model in HFD- and LFD-induced mice was collected after 4 weeks of SGF (62.5 mg/kg) treatment. An immunoblot showing the levels of AMPK activation represented by p-AMPK/AMPK and the expression of ferroptotic proteins in tumors. The blots (**H**) are representative of three repeats. The data (**I**–**L**) are expressed as means ± SD (*n* = 3). * *p* < 0.05, ** *p* < 0.01, and *** *p* < 0.001 vs. control.

It has been found that AMPK plays an inhibitory role in ferroptosis [36]. Meanwhile, the transcriptome analysis also suggested the strong involvement of the AMPK pathway in the antitumor effects of SGF in obesity-related CRC. These prompted us to perform a more detailed analysis of AMPK activation in vitro and in vivo. It was revealed that, as a hallmark of AMPK activation, AMPK phosphorylation, which mediates ACC phosphorylation, was substantially elevated in MC38 cells cocultured with mature adipocytes, consistent with what was reported previously [14]. Moreover, SGF repressed AMPK activation in MC38 cells induced by coculture with adipocytes but had no effect in the absence of adipocytes (Figure 5A–C). Likewise, SGF potently mitigated AMPK activation in the tumor tissue of HFD-induced mice, while it did not affect that of LFD-treated mice (Figure 5H,I). To further correlate AMPK activation with ferroptosis sensitivity in obesity-associated CRC, the expression of marker proteins for ferroptosis was examined by Western blotting. As expected, SGF was found to noticeably suppress the expression of GPX4, a critical antiferroptosis protein, in MC38 cells cocultured both with and without adipocytes (Figure 5D,F). Meanwhile, in both MC38 cells after coculture treatment and tumor tissue with HFD administration, the transferrin (TRF) expression was elevated, but this could be remarkedly reversed by SGF, indicating the involvement of iron metabolism in ferroptosis (Figure 5D,G,H,J). To our surprise, SGF exhibited no effect on the expression of SLC7A11, a key protein in the Xc-20 system and an upstream regulator of GPX4, in the MC38 cells and tumor tissue. Notably, the expression levels of SLC7A11 were significantly reduced in both MC38 cells after coculture with adipocytes for 48 h and in the tumor tissue of HFD-induced mice (Figure 5D,E,H,K). Acyl-CoA synthetase long-chain family member 4 (ACSL4), acting as a key downstream effector of AMPK in the regulation of ferroptosis, was further proven to be inhibited by HFD but evidently increased after SGF treatment. Together, these data strongly suggest that SGF could restrain the activation of the AMPK pathway to drive ferroptosis in obesity-associated CRC.

## 3. Discussion

SGF has been found to promote the formation of small lipid droplets and decrease the levels of cellular FFAs without affecting TG storage in adipocytes [31]. Its beneficial lipid-metabolic effects have been confirmed in vivo as well (Figure S3). These findings guided us to examine the antitumor actions of SGF in obesity-associated cancer. As expected, in the present study, SGF was proven to exert much stronger cytotoxic effects towards CRC cells cocultured with mature adipocytes compared to those without (Figure 1D–F and Figure S1B,C). Furthermore, HFD-induced obesity promoted both the growth of MC38 tumors and sensitivity to SGF (Figure 2B–D). These results suggest that SGF might be an excellent antitumor agent, particularly in obesity-related CRC, and obesity probably provides more effective targets for it.

Ferroptosis is a nonapoptotic form of programmed cell death that is induced by the overproduction of phospholipid hydroperoxides in an iron-dependent manner [37,38]. Accumulating evidence shows that ferroptosis plays an important role in CRC suppression. Metabolic and signaling pathways that regulate ferroptosis have been presented as potential therapeutic targets [39,40]. Recent studies have further linked ferroptosis to obesity-related cancers such as hepatocellular carcinoma and pancreatic ductal adenocarcinoma [41,42]. It also has been reported that HFD impairs ferroptosis, thereby aggravating colitis-associated carcinogenesis [34]. Consistently, our transcriptome analysis of MC38 tumors hinted at the involvement of ferroptosis in high-fat-induced and SGF-treated CRC (Figure 3B–D), and the cell viability assay confirmed that SGF induced ferroptotic cell death in MC38 cells under an adipocyte environment (Figure 4C). Moreover, FFAs could induce ferroptosis resistance in MC38 cells, as aforementioned, while SGF dramatically sensitized MC38 cells to ferroptosis, albeit in the presence of FFAs (Figure 4D). On this basis, ferroptosis induced by SGF was further substantiated by different approaches in vitro (Figure 4E–I). SGF had no effect on ferroptosis in MC38 cells without adipocyte coculture (Figure 4B), suggesting that the specific regulation of ferroptosis is probably responsible for its high cytotoxic efficacy in obesity-associated CRC.

AMPK, a critical sensor of the cellular energy status, has been proven to play a role in tumor biology, but its exact role is highly context-dependent [43]. While the tumor-suppressive role of the AMPK pathway is well established [44], multiple studies have convincingly indicated a tumor-promoting role of AMPK in some tumor types [45,46,47,48]. Recent studies have linked AMPK to ferroptosis, showing that AMPK possesses negative regulatory effects on ferroptosis [49,50]. Moreover, a study has proposed that the presence of adipocytes could enhance AMPK activation in CRC cells [14]. These findings indicate that AMPK’s pro-tumor function might be partly mediated through its inhibition of ferroptosis, at least in a high-fat context. Our transcriptome analysis also suggested the engagement of the AMPK pathway in the bio-process of SGF-treated MC38 tumors in obese mice (Figure 3B). In light of this, we supposed that the activation of the AMPK pathway, acting as an upstream signal of ferroptosis, might mediate the cytotoxic effects of SGF in obesity-related CRC. The Western blotting assay indeed confirmed the activation of the AMPK pathway in both MC38 cells cocultured with adipocytes and the tumor tissue of obese mice. More importantly, either in vitro or in vivo, SGF could specifically inhibit the activation of the AMPK pathway in CRC under a high-fat context (Figure 5A–C,H,I). Meanwhile, the expression of critical proteins in the ferroptosis pathway was determined. For the cystine-metabolic process, antiferroptosis protein GPX4 was significantly decreased after SGF treatment, while SLC7A11, as its upstream regulator, was not affected by SGF (Figure 5D–F). This might have been due to the low basal expression of SLC7A11 in both MC38 cells cocultured with adipocytes and the tumor tissue of obese mice (Figure 5D,E,H,K), in contrast to previous reports showing that HFD could exacerbate lung cancer progression by upregulating SLC7A11 [51]. Meanwhile, it has been proposed that, while the AMPK activation status did not correlate with ferroptosis sensitivity in SLC7A11-high cells, a direct inverse correlation between AMPK activation and ferroptosis sensitivity in SLC7A11-low cells could be observed [36]. Accordingly, AMPK activation based on the low expression of SLC7A11 altering the cellular sensitivity to ferroptosis under HFD treatment was mechanistically responsible for the distinctive antitumor activity of SGF in obesity-related CRC. It is known that ACSL4, which mediates the biosynthesis of polyunsaturated fatty acid (PUFA)-containing lipids, acts as a downstream event in AMPK activation in ferroptosis inhibition [36,52]. A recent study reported that HFD could impair ferroptosis via downregulating ACSL4 [53]. In line with these, we found that, contrary to AMPK activation, the protein expression of ACSL4 was almost blocked by HFD, while it markedly rebounded after SGF treatment (Figure 5H,L). These results indicate that AMPK mediated the cytotoxic effect of SGF against obesity-related CRC as a tumorigenesis gene at least partially through ferroptosis induction. In the present study, our data also showed that TRF increased remarkably in the high-fat context and SGF abated this increase (Figure 5D,G,H,J), indicating that the ferroptosis induced by SGF might involve iron homeostasis as well. Future studies will further dissect the potential molecular mechanism of AMPK-mediated ferroptosis induced by SGF in obesity-associated CRC development.

**Figure 6:**
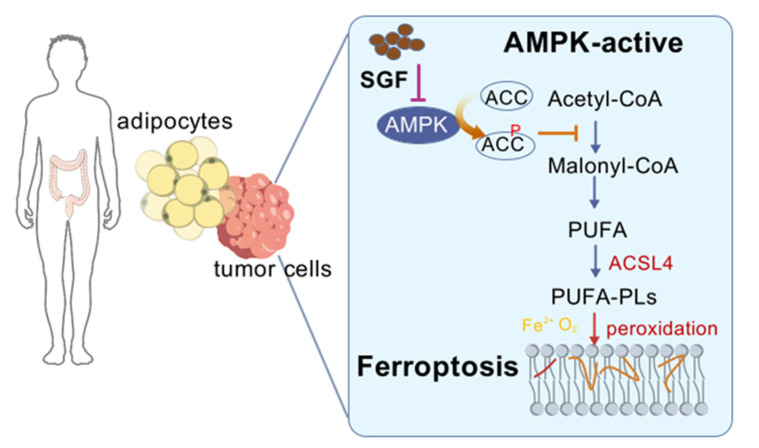
Schematic representation of the mechanism by which SGF exerts cytotoxic effects against obesity-associated CRC. Created with BioGDP.com.

This study demonstrates that SGF possesses distinctive antitumor activity against obesity-associated CRC in vitro and in vivo. Mechanistically, SGF exhibited cytotoxic effects against MC38 cells through inhibiting AMPK activation, thereby driving ferroptosis, specifically in a high-fat context (Figure 6). Our data expand the current knowledge of the obesity paradox in cancer therapy and emphasize the antitumor effects of SGF towards obesity-related CRC for the first time. This will provide new therapeutic ideas for obesity-associated cancer.

However, this study primarily relied on a single cell line for most experiments, which may limit the reliability and generalizability of the results. In future studies, we will validate the conclusions using additional authenticated colorectal cancer lines. Moreover, this study did not identify which flavonoids of *Smilax glabra* have the greatest impact on the antiproliferative effects in MC38 colon adenocarcinoma cells. This is also a limitation of this study, and future investigation of this aspect would be of great interest, as it could provide valuable insights into the underlying mechanisms and potential therapeutic applications.

## 4. Materials and Methods

### 4.1. Regents

3-(4,5-Dimethylthiazol-2-y1)-2,5-dipheny-ltetrazolium bromide (MTT), 3-isobutyl-1-methylxanthine (IBMX), and oleic acid were purchased from Sigma-Aldrich (Cat. Nos. M2128, I7018, and O1383, St. Louis, MO, USA). Dexamethasone (Dex) was obtained from Sango (Cat. No. A601187, Shanghai, China), and insulin was obtained from Shanghai Yuanye BioTechnology Co., Ltd. (Cat. No. S31559, Shanghai, China). The cell culture medium, trypsin, penicillin–streptomycin, and fetal bovine serum (FBS) were from Gibco (Burlington, MA, USA). Oil Red O was obtained from Solarbio (Cat. No. G1260, Beijing, China). Malondialdehyde (MDA) assay kits were obtained from Dojindo (Cat. No. M496, Kumamoto, Japan). The glutathione assay kit and reactive oxygen species (ROS) assay kit were bought from Beyotime (Cat. Nos. S0053 and S0033, Nanjing, China). The Insulin ELISA kit was purchased from Millipore (Cat. Nos. RAB0817, Millipore, Bedford, MA, USA). Primary antibodies against SLC7A11/xCT (26864-1-AP), TRF (17435-1-AP), and GPX4 (14432-1-AP) were purchased from Proteintech (Rosemont, IL, USA). Anti-AMPKα (D63G4) (5832), anti-phospho-AMPKα (Thr172) (40H9) (2535), anti-ACC (C83B10) (3676), and anti-phospho-ACC (Ser79) (D7D11) (11818) were obtained from Cell Signaling Technology (Danvers, MA, USA). ACSL4 was from Abcam (ab155282, Cambridge, MA, USA) and β-actin was from HUABIO (EM21002, Shanghai, China). BODIPYTM 581/591 C11 was obtained from Thermo Fisher Scientific (Cat. No. D3861, Waltham, MA, USA). Ferrostatin-1 and Erastin were purchased from MedChemExpress Technology (Cat. Nos. HY-100579 and HY-15763, Monmouth Junction, NJ, USA).

### 4.2. Smilax glabra Flavonoid Preparation

SGF was prepared from the rhizomes of *Smilax glabra* (Zhejiang Chinese Medical University Medicine Ltd., Hangzhou, China), as described previously [28]. Briefly, the rhizome of *Smilax glabra* was extracted with 60% ethanol twice at a solid–liquid ratio of 1:20 (*w/v*). After filtration, the extractions, suspended in 10% ethanol, were loaded onto a HP700 macroporous resin column and eluted with 60% ethanol at a flow rate of 1 mL/min. The eluted fractions, consisting mainly of total flavonoids, were concentrated using a rotary vacuum evaporator at 45 °C and then freeze-dried. The content of flavonoids in SGF was determined to be 98.65% by UV spectrophotometry and calculated as rutin equivalents. The high-performance liquid chromatography (HPLC) (Waters Alliance e2695, Waters, Milford, MA, USA) quantitative analysis of astilbin was undertaken for the quality control of SGF. The percentage content of astilbin in the dry powder of SGF was 25.13% (Figures S4 and S5).

### 4.3. Cell Culture and Differentiation

Mouse 3T3-L1 pre-adipocytes, murine colon carcinoma CT26 cells, and human colorectal cancer HCT116 cells were provided by the National Collection of Authenticated Cell Cultures (Shanghai, China). Murine colorectal adenocarcinoma MC38 cell lines were purchased from MingzhouBio (Ningbo, China). The cells were routinely cultured in DMEM (high glucose), McCoy’5a, or RPMI medium containing 10% neonatal calf serum (CS) (TIANHANG Biotechnology, Huzhou, China), 100 IU/mL penicillin, and 100 μg/mL streptomycin in an incubator at 37 °C with a humidified atmosphere of 5% CO_2_. The cell line identity was validated by short tandem repeat profiling, and routine mycoplasma testing was negative for contamination.

For adipocyte differentiation, 3T3-L1 cells were maintained in high-glucose DMEM with 10% CS at confluence for two days (day 0), after which the cells were stimulated with DMI differentiation medium consisting of 1 μM Dex, 0.5 mM IBMX, and 10 μg/mL insulin for two days (day 2). The cells were then maintained in growth medium containing 10 μg/mL insulin for two more days (day 4). Subsequently, the cells were switched to fresh growth medium every other day for an additional two or four days to differentiate to mature adipocytes [31].

### 4.4. Mice and Dietary Treatment

C57BL/6J mice were purchased from Sino-British SIPPR/BK Laboratory Animal Co., Ltd. (Shanghai, China) and housed in a specific pathogen-free (SPF) animal facility and maintained by the Laboratory Animal Research Center of Zhejiang Chinese Medical University. All mouse procedures were carried out under Institutional Animal Care and Use Committee (IACUC)-approved protocols from Zhejiang Chinese Medical University (IACUC-20190923-15). For the dietary intervention, male C57BL/6J mice starting at 6–8 weeks old were randomly assigned to cages (5 mice per cage) to receive a high-fat diet (HFD, 60% kcal from fat, Research Diets PD6001) or a control low-fat diet (LFD, 10% kcal from fat). The mice were maintained on their respective diets for 14 weeks in total [42]. Metabolic parameters were assessed at 10 and 14 weeks of dietary treatment. After a 10 h fast, the concentrations of blood glucose, triglycerides (TG), and total cholesterol (TC) were measured with an automatic biochemistry analyzer (Hitachi 7020; Hitachi, Tokyo, Japan). Collected blood was centrifuged at 3000 rpm for 10 min at 4 °C, and plasma was aliquoted and stored at −80 °C until analysis. Plasma insulin (Millipore) concentrations were analyzed using a commercial ELISA kit according to the manufacturer’s instructions. The homeostatic model assessment for insulin resistance (HOMA-IR) was calculated using the following formula: fasting insulin in µU/mL multiplied by fasting glucose in mmol/L and divided by 22.5 [54].

### 4.5. Oil Red O Lipid Staining

Oil Red O (ORO) staining was performed as previously reported, with minor modifications [31]. Briefly, differentiated adipocytes were fixed in 4% paraformaldehyde for 30 min and subsequently permeabilized with a solution of 0.1% TritonX-100 for 10 min at room temperature. Then, after repeated washes, the cells were stained with a freshly prepared ORO working solution (isopropanol/water 3:2, *v*/*v*) for 20 min. Lipid droplets (LD) was visualized by Zeiss Axiovert phase-contrast microscopy (Oberkochen, Germany) at magnification of 40×.

### 4.6. Conditioned Medium (CM) Collection and Transwell Coculture

For CM collection, MC38 cells or the mature adipocytes were treated with serum-free medium for 24 h, and the culture supernatants were collected, centrifuged at 1500× *g* for 5 min, and filtered through a 0.22 µm filter. For the two-step CM transfer experiments, mature adipocytes were exposed to fresh media mixed with CM from MC38 cells at a ratio of 1:1 (*v*/*v*) for 24 h. Then, the CM was replaced with fresh culture medium, and, 24 h later, the supernatants from the adipocytes (i.e., adipocyte-CM) were collected, centrifuged as above, and filtered using a 0.22 µm filter and then mixed with fresh culture medium at a 1:1 ratio and applied to treat MC38 cells for 24 h for in vitro proliferation assays.

For the Transwell coculture experiments, MC38 tumor cells were seeded in the top chamber of the Transwell system (0.4 μM pore size; BD Bioscience, San Jose, CA, USA) for 24 h of adherence and cocultivated with or without mature adipocytes in the bottom chamber for the indicated time. The incubation of the cells in the Transwell system allowed the diffusion of soluble factors.

### 4.7. Cell Viability Assay

MC38 cells were seeded at 1.0 × 10^4^ cells/well in a 96-well plate and incubated at 37 °C in a humidified atmosphere with 5% CO_2_. After 24 h, the media were changed to fresh media mixed with CM (from 3T3-L1 fibroblasts or differentiated mature adipocytes) at a ratio of 1:1 (*v*/*v*) in the presence or absence of SGF at the indicated concentrations and incubated for another 24 h. For the inhibition assay, the confluent MC38 cells were exposed to the two-step CM to mimic the putative bidirectional communication between tumor cells and adipocytes in the obese tumor microenvironment. The cells were pre-treated simultaneously with Ferrostatin-1 (Fer-1) for 60 min, followed by co-incubation with the tested compounds for an additional 48 h. Each concentration was repeated for four wells. Four hours before the end, the extent of cell proliferation was detected using the MTT assay and the absorbance was measured at 570 nm with a microplate reader (Bio-Rad, Hercules, CA, USA) [31].

### 4.8. Cell Migration Assay

Transwell migration assays were performed by following previously described procedures [55]. Briefly, MC38 cells were cocultured with or without adipocytes in the presence or absence of 0.125 mg/mL SGF for 72 h and subsequently subjected to Transwell migration assays using 20% FBS in DMEM as the chemoattractant. A total of 200,000 cells were seeded into Transwells and allowed to migrate for 8 h. Subsequently, the non-migrating cells on the upper side of the membrane surface were wiped off with a cotton swab, while the migrating cells were fixed in 4% paraformaldehyde for 2 min, followed by washing with PBS three times. The membrane was then stained with 0.5% crystal violet solution for 5 min, followed by washing with PBS three times. The membrane was air-dried and then photographed with a light microscope (Axiover200, Zeiss, Oberkochen, Germany). The number of migrated cells was quantified by ImageJ 1.40g.

### 4.9. RNA Sequencing and Data Analysis

Tumor samples from each group of mice were subjected to RNA sequencing. Total RNA was isolated and purified using the TRIzol reagent (Invitrogen, Carlsbad, CA, USA), according to the manufacturer’s instructions. The RNA integrity was assessed with a Bioanalyzer 2100 (Agilent, Santa Clara, CA, USA) with RIN number > 7.0 and confirmed by electrophoresis with a denaturing agarose gel. RNA sequencing was performed using the Illumina NovaSeq™ 6000 platform (LC-Bio Technology CO., Ltd., Hangzhou, China), generating 2 × 150 bp paired-end reads. The Cutadapt software (https://cutadapt.readthedocs.io/en/stable/, accessed on 22 July 2020) was used to remove reads containing adaptor contamination. The reads of all samples were aligned to the mouse mm10 reference genome using the HISAT2 software (https://daehwankimlab.github.io/hisat2/, accessed on 22 July 2020). The mapped reads from each sample were assembled using StringTie (http://ccb.jhu.edu/software/stringtie/, accessed on 22 July 2020) with the default parameters. StringTie and ballgown (http://www.bioconductor.org/packages/release/bioc/html/ballgown.html, accessed on 22 July 2020) were used to estimate the expression levels of all transcripts and determine the expression levels of mRNAs by calculating the FPKM. The differentially expressed genes (DEGs) were selected using the DESeq2 software version 3.20 with a cut-off of *p*-value < 0.05 and absolute fold change > 1.5. Gene Ontology (GO) annotation and Kyoto Encyclopedia of Genes and Genomes (KEGG) pathway enrichment analysis were used to analyze the main functions of the DEGs according to the NCBI Gene Ontology database. An adjusted *p*-value < 0.05 was set as the threshold for significantly enriched GO terms and KEGG pathways.

### 4.10. Fatty Acid-Dependent Sensitization of Ferroptosis

MC38 cells were plated onto 96-well plates at a density of 1 × 10^4^ per well. After overnight incubation, the cells were treated or left untreated with 0.1 mM oleic acid or fatty acids + 2 μM Ferrostatin-1 for 60 min. Subsequently, the cells were treated simultaneously with fatty acids with 2 μM Erastin, a specific ferroptosis inducer, in the presence or absence of 0.125 mg/mL SGF for 48 h, and the cell viability was evaluated using the MTT assay [34,36].

### 4.11. ROS and Lipid Peroxidation Staining

After Transwell coculture, the MC38 cells were digested with trypsin and collected. The cells were incubated for 30 min at 37 °C with DCFH-DA for ROS detection or C11-BODIPY 581/591 (Invitrogen) for lipid peroxidation assessment [34,56]. The ROS and lipid peroxidation levels were imaged using fluorescence microscopy or analyzed on a flow cytometer (CytoFLEX, Beckman Coulter, Brea, CA, USA) using the CytExpert 2.4 software.

### 4.12. Malondialdehyde (MDA) Assay

As a major indicator of lipid peroxidation, the content of MDA in the cells was detected with an MDA assay kit (Dojindo Laboratories, Kumamoto, Japan, M496), according to the manufacturer’s instructions. Briefly, cells were homogenized in lysis buffer, incubated with thiobarbituric acid at 95 °C for 15 min, and centrifuged, and the supernatant was evaluated on a fluorescence microplate reader (Varioskan Flash, ThermoFisher, Waltham, MA, USA). Then, the MDA content was calculated using the standard calibration curve supplied in the kit, normalized to the protein concentration, and expressed as nmol/mg protein.

### 4.13. Glutathione Assay

A glutathione assay kit (Beyotime, S0053, Haimen, China) was used to measure the content of GSH [34]. The cells were collected, washed twice with ice-cold PBS, lysed by freezing and thawing them twice in liquid nitrogen at 37 °C, and centrifuged. The supernatant was collected and used for the assays, referring to the manufacturer’s protocols. GSH and oxidized GSH (GSSG) standard solutions and samples were loaded into 96-well plates. Next, a buffer solution was added to each well, and the plate was incubated at 25 °C for 5 min. A NADPH solution was then added to each well, the plate was incubated at 25 °C for 30 min, and the absorbance was read at 412 nm using a microplate reader. The content of GSSG was subtracted from the amount of total glutathione (GSSG + GSH) to calculate the content of GSH, which was then normalized to the total protein level in each sample.

### 4.14. Tumor Xenograft Model

After 10 weeks of dietary treatment, mice were injected subcutaneously in the right flank with 6 × 10^5^ MC38 cells [57]. Once palpable tumors were present, LFD and HFD mice were both randomly assigned to receive oral administration with either 62.5 mg/kg SGF or the same volume of saline once daily for four weeks.

Body weights and tumor sizes were measured every two days. The tumor size was measured using a digital slide caliper and volumes (mm^3^) were calculated as follows: tumor volume (mm^3^) = L × W^2^/2, where L is the length of the tumor and W is the width of the tumor. Mice were euthanized with a lethal dose of CO_2_ inhalation after the final drug administration and tumors were carefully excised and weighed.

### 4.15. Western Blotting

Total protein from cells or tissues was harvested with ice-cold RIPA buffer plus a protease inhibitor and further quantified using a BCA Protein Assay Kit (KeyGEN BioTECH, Nanjing, China). Equivalent amounts of protein were separated on 10% SDS-PAGE and transblotted onto a 0.22 μm PVDF membrane (Bio-Rad, Hercules, CA, USA). Membranes were blocked with 5% skimmed milk (*w*/*v*) in TBST buffer for 1 h and immunoblotted with the indicated primary antibodies with gentle rocking overnight at 4 °C. Subsequently, they were further incubated with the corresponding secondary antibodies (HUABIO, 1:1000) for 1.5 h at room temperature. Finally, specific protein bands were visualized and quantified using the Odyssey Infrared Imaging System (Li-Cor Biosciences, Lincoln, NE, USA). Data normalization was performed using the bands of β-actin (1:1000 dilution).

### 4.16. Statistical Analyses

Data were presented as means ± SD and examined for their statistical significance with an analysis of variance (ANOVA) and Student’s *t*-test after a normality test. The analyses and graphs were performed using the GraphPad Prism 8.0 software (GraphPad Software, San Diego, CA, USA). *p*-values of less than 0.05 were considered statistically significant.

## Data Availability

All data are available as Supplementary Materials.

## Supplementary Materials

The following supporting information can be downloaded at: https://www.mdpi.com/article/10.3390/ijms26062476/s1.
