# Supplementary material for: Smilax glabra Flavonoids Inhibit AMPK Activation and Induce Ferroptosis in Obesity-Associated Colorectal Cancer
Source: Int J Mol Sci. 2025 Mar 10;26(6):2476. doi: 10.3390/ijms26062476 (PMC11942472; doi:10.3390/ijms26062476)
Supplement: Supplementary file 1 [file ijms-26-02476-s001.zip › ijms-3421809-supplementary.pdf]

International Journal of Molecular Sciences

*Supplementary Materials*

# *Smilax glabra* Flavonoids Inhibit AMPK Activation and Induce Ferroptosis in Obesity-Associated Colorectal Cancer

**Jianqin Xu [1], Zhaowei Cai [1,2], Ziyao Pang [1,2], Jiayan Chen [1,2], Keyan Zhu [1], Dejun Wang [1,2,*] and Jue Tu [1,*]**

[1] Laboratory Animal Research Center, Academy of Chinese Medical Sciences, Zhejiang Chinese Medical University, Hangzhou 310053, China; 13958083242@163.com (J.X.); zwcai@zcmu.edu.cn (Z.C.); pang3056527363@163.com (Z.P.); 18407878185@163.com (J.C.); zjzygyz@163.com (K.Z.)

[2] School of Pharmacy, Zhejiang Chinese Medical University, Hangzhou 310053, China

* Correspondence: wdj@zcmu.edu.cn (D.W.); tujue@zcmu.edu.cn (J.T.); Tel.: +86-570-8661-3662 (D.W.); +86-570-8661-3782 (J.T.)

## Supplementary Figures

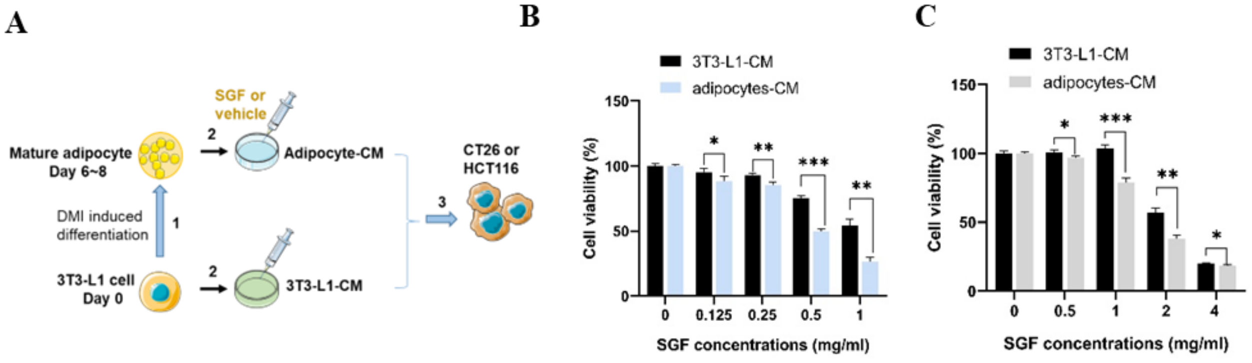


**Figure S1.** SGF inhibited the proliferation of CT26 and HCT116 cells in the adipose microenvironment. (**A**) Flowchart of conditioned medium (CM) preparation and treatment of tumor cells, as described in Figure 1. (**B**–**C**) The cell viabilities of CT26 (**B**) and HCT116 (**C**) cells treated with SGF at the indicated concentrations for 24 h in the corresponding CM via MTT assay. Data are expressed as mean ± SD for three independent experiments. * $p < 0.05$, ** $p < 0.01$, *** $p < 0.001$ vs. 0 mg/ml.

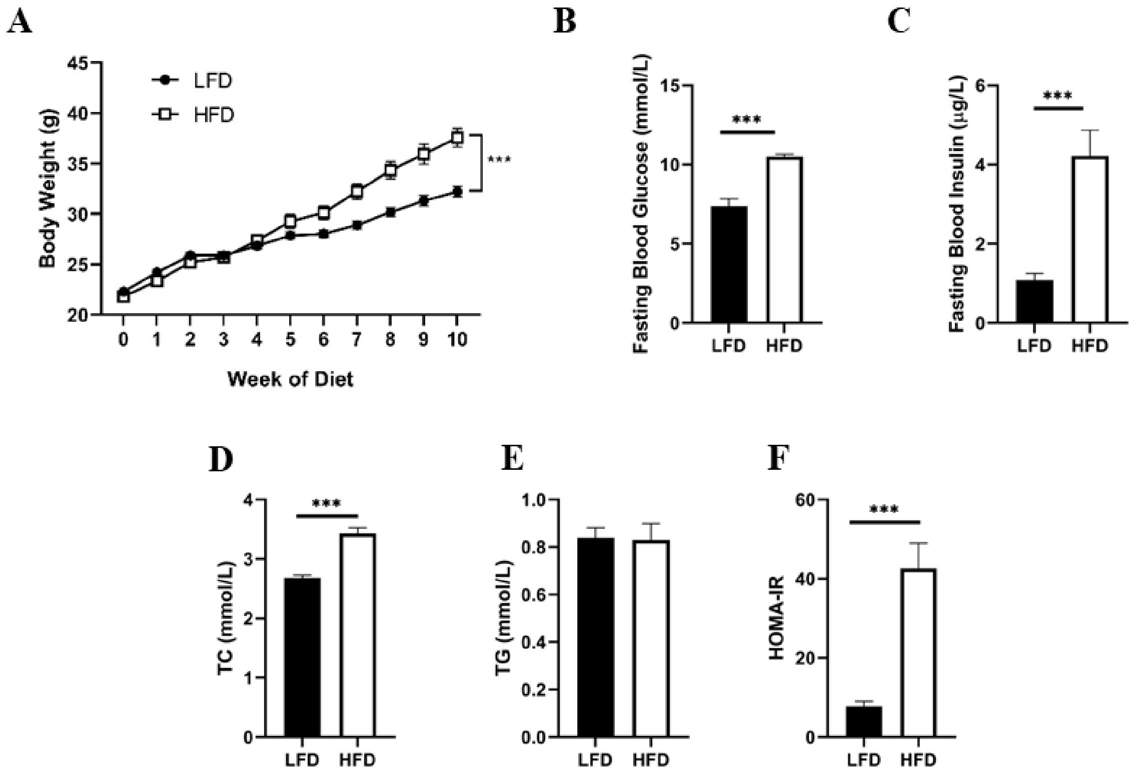


**Figure S2.** High Fat Diet feeding induces an obese phenotype. (**A**) Mouse body weights during 10 weeks of LFD or HFD feeding prior to MC38 tumor injection (n = 12 mice per diet). (**B**–**F**) Blood glucose (**B**), blood insulin (**C**), TC (**D**) and TG (**E**) were measured following a 10-h fast after 10 weeks of diet treatment (n = 6 mice per diet). HOMA-IR (**F**) was calculated. The data were expressed as means ± SEM. *** $p < 0.001$ vs LFD.

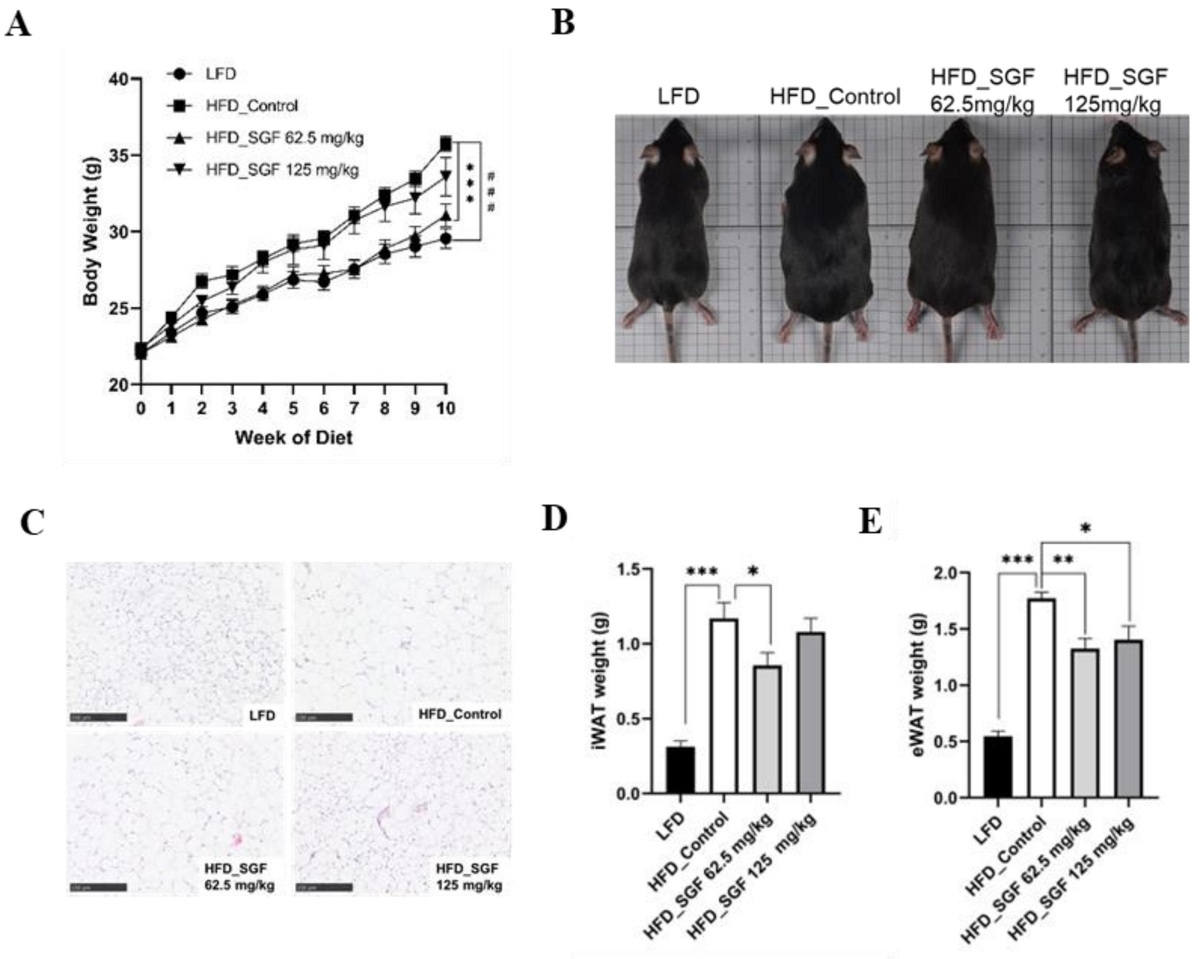


**Figure S3.** SGF exerted beneficial lipid metabolic actions in obese mice induced by high-fat diet. (**A**) The C57BL/6 mice were treated by LFD, HFD or HFD supplemented with SGF (62.5 mg/kg, 125 mg/kg) for 10 weeks and the body weights were recorded each week. (**B**) Represent images of mice after 10 weeks of different treatment as indicated. (**C**) Histology of inguinal adipose tissue (IAT). Representative hematoxylin-eosin-stained IAT sections (original magnification ×100). (**D**,**E**) Wet weights of inguinal adipose tissue (iWAT) and epididymal adipose tissue (eWAT) of mice in different groups. The data were expressed as means ± SEM (n = 8). * $p < 0.05$, ** $p < 0.01$, *** $p < 0.001$ vs HFD_Control and ### $p < 0.001$ vs. LFD.

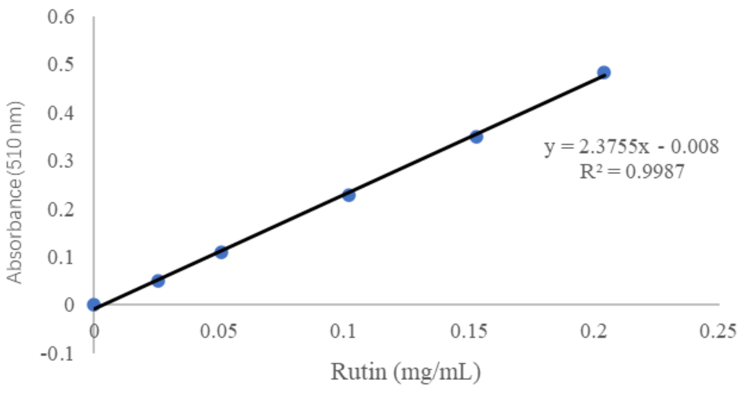


**Figure S4.** Standard curve of rutin. The total flavonoid content of SGF was measured using a colorimetric assay. A known volume of the extract or standard solution of rutin was added to a 10 mL volumetric flask. Ethanol (70%, v/v) was added to make a volume of 2 mL. Firstly, 0.3 mL of $NaNO_2$ (5%, w/v) was added to the flask. After 6 min, 0.3 mL of Al(NO3)3 (10%, w/v) was added, and after

6 min, 4 mL of NaOH (1.0 M) was added to the mixture followed by the addition volume of distilled water. Absorbance was read at 510 nm against the blank. The content of flavonoids was calculated as rutin equivalents.

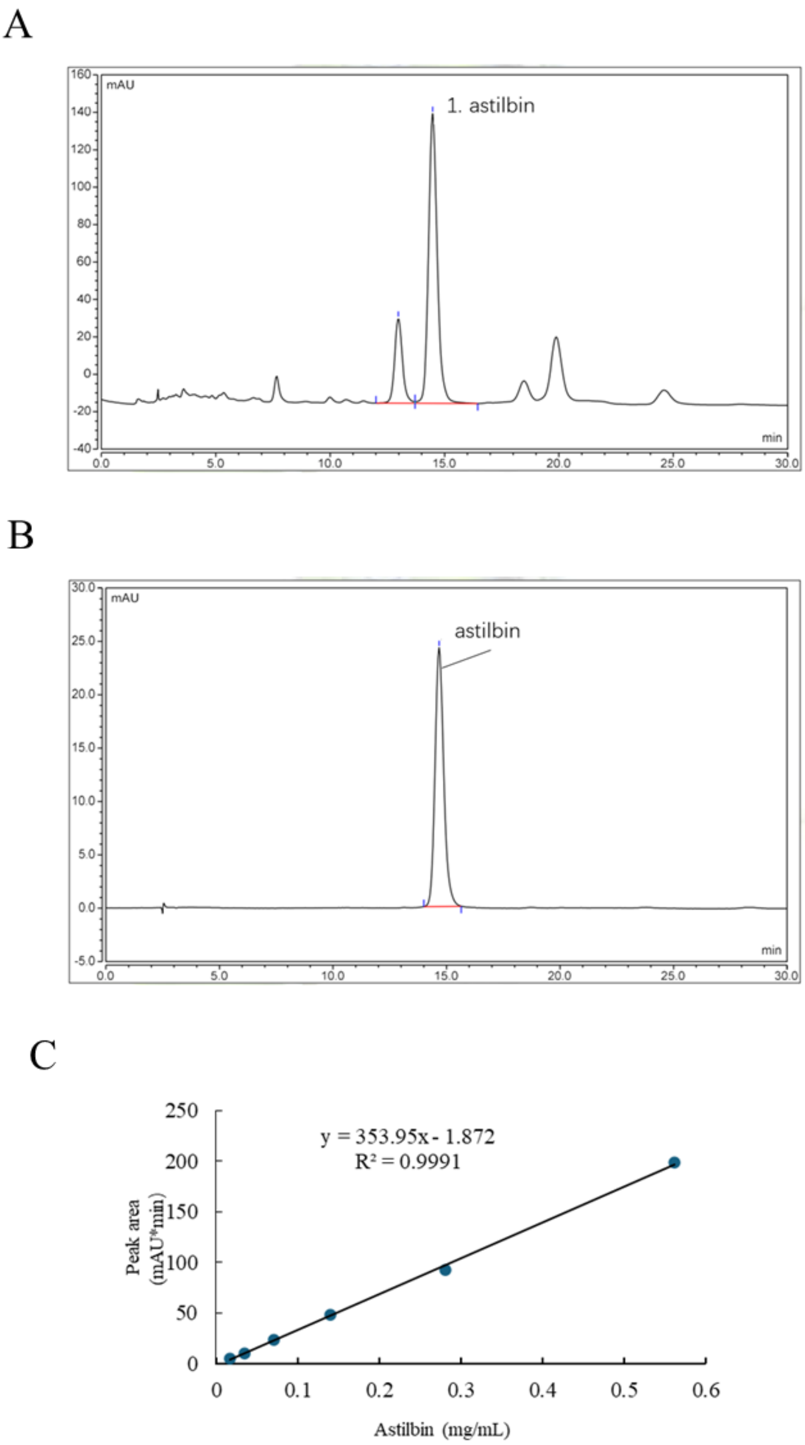


**Figure S5.** HPLC analysis of SGF. The content of astilbin in SGF was calculated from the external calibration curve of standard. HPLC chromatograms of (**A**) SGF and (**B**) the astilbin standard compound; (**C**) Standard curve of astilbin.
